# Mechanical Exfoliation of Expanded Graphite to Graphene-Based Materials and Modification with Palladium Nanoparticles for Hydrogen Storage

**DOI:** 10.3390/nano13182588

**Published:** 2023-09-19

**Authors:** Darren Chow, Nicholas Burns, Emmanuel Boateng, Joshua van der Zalm, Stefan Kycia, Aicheng Chen

**Affiliations:** 1 Electrochemical Technology Center, Department of Chemistry, University of Guelph, 50 Stone Road East, Guelph, ON N1G 2W1, Canada; dchow07@uoguelph.ca (D.C.); eboate01@uoguelph.ca (E.B.); vanderzj@uoguelph.ca (J.v.d.Z.); 2 Department of Physics, University of Guelph, 50 Stone Road East, Guelph, ON N1G 2W1, Canada; burnsn@uoguelph.ca

**Keywords:** hydrogen storage device, graphene-based nanocomposites, liquid phase exfoliation, high shear mixing, probe tip sonication, palladium nanoparticle

## Abstract

Hydrogen is a promising green fuel carrier that can replace fossil fuels; however, its storage is still a challenge. Carbon-based materials with metal catalysts have recently been the focus of research for solid-state hydrogen storage due to their efficacy and low cost. Here, we report on the exfoliation of expanded graphite (EG) through high shear mixing and probe tip sonication methods to form graphene-based nanomaterial ShEG and sEG, respectively. The exfoliation processes were optimized based on electrochemical capacitance measurements. The exfoliated EG was further functionalized with palladium nanoparticles (Pd-NP) for solid-state hydrogen storage. The prepared graphene-based nanomaterials (ShEG and sEG) and the nanocomposites (Pd-ShEG and Pd-sEG) were characterized with various traditional techniques (e.g., SEM, TEM, EDX, XPS, Raman, XRD) and the advanced high-resolution pair distribution function (HRPDF) analysis. Electrochemical hydrogen uptake and release (Q_H_) were measured, showing that the sEG decorated with Pd-NP (Pd-sEG, 31.05 mC cm^−2^) and ShEG with Pd-NP (Pd-ShEG, 24.54 mC cm^−2^) had a notable improvement over Pd-NP (9.87 mC cm^−2^) and the composite of Pd-EG (14.7 mC cm^−2^). Q_H_ showed a strong linear relationship with an effective surface area to volume ratio, indicating nanoparticle size as a determining factor for hydrogen uptake and release. This work is a promising step toward the design of the high-performance solid-state hydrogen storage devices through mechanical exfoliation of the substrate EG to control nanoparticle size and dispersion.

## 1. Introduction

The reliance of fossil fuels as a fuel source has had clear effects on environmental pollution and threatens human health due to the emission of greenhouse gases. An attractive alternative fuel carrier is hydrogen, which only produces water as a product of its combustion [1]. In addition, hydrogen has a remarkable gravimetric energy density of 142 MJ/kg, which is larger than other fuels such as gasoline, which has a gravimetric energy density of 47 MJ/kg [2]. One challenge with the use of hydrogen as a fuel carrier is its storage. This is due to hydrogen being gaseous at room temperature and having a low volumetric energy density. The current traditional methods of storage for hydrogen include pressurized gas and cryogenic compression which both have intrinsic issues such as safety concerns, low volumetric density, and boil off [3]. However, materials-based storage processes involving physical adsorption onto a material constitute a more attractive storage method that has been under the recent focus of research [4,5,6].

In recent years, the use of nanomaterials has been efficacious in various energy applications as well as hydrogen storage applications [7,8]. Carbon-based materials and nanomaterials such as graphene [9,10], graphitic carbon [11], carbon nanotubes [12,13], and activated carbon [14] are all of particular interest to hydrogen storage applications. Graphene specifically has unique properties including low cost, lightweight, fast reaction kinetics, and high specific surface area [10,15]. To further improve the hydrogen sorption abilities of graphene, various metal nanoparticles such as Pd, Ni, and Ti have been used due to the hydrogen spillover mechanism [9,10,16]. The spillover mechanism involves the sorption of dissociated hydrogen onto the metal nanoparticles followed by migration and diffusion onto the supporting material [17]. Of particular interest is the use of Pd due to its high affinity for hydrogen sorption as well as its low cost compared to other metallic catalysts [18]. A common approach therefore would include the use of Pd-decorated graphene nanomaterials and nanocomposites [19]. The Pd acts to absorb high amounts of hydrogen, which diffuses as atomic hydrogen onto the supporting graphene-based nanomaterials via the spillover process.

The use of high shear force mixing and sonication for the liquid phase exfoliation (LPE) of graphite has recently been the focus of research to prepare graphene and improve the properties of the prepared graphene [20,21,22,23,24]. LPE has attracted considerable research interest due to several advantages. Firstly, in traditional chemical exfoliation techniques, such as Hummer’s method, a large amount of harsh acids is utilized, which causes environmental issues [25]. In contrast, the LPE techniques are commonly conducted in aqueous solutions without the need for use of acids [24,26,27,28]. Furthermore, chemical methods introduce oxygen functional groups and defects in the graphene sheet, which may reduce the electrochemical activity of the formed graphene-based materials [29,30]. To overcome this issue, a reduction step is often required to reduce the oxygen functional groups, which is not needed in LPE methods as they do not create oxygen functional groups. Another reason that LPE has been the focus of research is the scalability and simplicity [20].

In the case of sonication, two separate mechanisms for the exfoliation of graphite are involved. The primary mechanism involves cavitation caused by the ultrasonic vibration, acting upon the graphite layers and generating tensile stress followed by exfoliation. The secondary mechanisms include the wedge effect, where a microjet is produced acting as a wedge driven between graphite layers causing exfoliation, and a shear effect caused by opposing forces acting on adjacent sheets [31]. Meanwhile, the high level of shear forces generated in high-shear mixing is from the low clearance between the rotor and stator of roughly 100 μm. For the exfoliation of graphite, the opposing shear forces acting on individual graphite sheets cause exfoliation, separating the sheets laterally. The use of LPE may facilitate a more uniform distribution and the formation of smaller nanoparticles [32].

Commonly, one method for solid-state hydrogen storage is the use of physisorption onto materials with the aid of high pressures. As extreme high pressures are required for hydrogen storage which also commonly include extreme low temperatures, notable energy requirements give poor efficiency generally reporting less than 1 wt.% hydrogen storage under ambient conditions [5]. In contrast, an alternative method of solid-state hydrogen storage utilizes electrochemistry as the method for hydrogen sorption. The use of electrochemistry has been an attractive method due to the ability for in situ hydrogen uptake, where up to 2.2 wt.% has been reported under ambient conditions [33].

This work reports on the effects of the high shear force mixing and the probe sonication on the exfoliation of expanded graphite (EG) as a pretreatment for the deposition of Pd nanoparticles (Pd-NP). The synthesized nanomaterials and nanocomposites were characterized through several techniques including scanning electron microscopy (SEM), transmission electron microscopy (TEM), energy-dispersive X-ray spectroscopy (EDX), X-ray photoelectron spectroscopy (XPS), X-ray diffraction crystallography (XRD), high-resolution pair distribution function (HRPDF) analysis, Raman spectroscopy, and various electrochemical techniques. To the best of our knowledge, the use of high shear mixing and probe sonication prior to the deposition of Pd for the use in hydrogen storage has not been previously reported.

## 2. Materials and Methods

### 2.1. Materials

Graphite was obtained from Zentek Ltd. (Guelph, ON, Canada). Phosphoric and sulfuric acid were purchased from Fisher Scientific. Iron (III) chloride hexahydrate, potassium permanganate, palladium (II) nitrate, sodium borohydride, and Nafion perfluorinated resin solution (5 wt% in lower aliphatic alcohols and water) were all used as received from Sigma-Aldrich (St. Louis, MO, USA). Ultrapure argon gas was also used as received from Linde Canada. Purified water (18.2 MΩ) was obtained by a ThermoFisher Scientific (San Diego, CA, USA) Barnstead NANOpure Diamond UV ultrapure water system. Powdered diamond nanoparticles were obtained from Sigma-Aldrich for the calibration in the HRPDF analysis.

### 2.2. Synthesis of EG

EG was prepared as previously described [19]. Briefly, using an ice–water bath for cooling, 40 mL of a 4:1 *v*/*v* acidic solution of phosphoric and sulfuric acid was added to 1 g of graphite powder and was stirred using a magnetic stir bar for 5 min. Then, 4 g of potassium permanganate was added while mixing. After 30 min, 0.5 g of iron (III) chloride was added, and the solution was left to stir for an additional 1 h. The solution was then centrifuged and rinsed with purified water. The obtained sedimented graphite intercalated compound (GIC) was left to dry completely in an oven at 60 °C. The GIC was crushed with a mortar and pestle, loaded into a ceramic crucible, covered with a steel lid, and thermally expanded at 600 °C for 5 min to produce EG.

### 2.3. Synthesis of Mechanically Exfoliated Nanomaterials

To prepare the mechanically exfoliated materials, a 4 mg/mL suspension of EG was prepared in a solvent ratio of 30% *v*/*v* EtOH in pure water. The experiments were conducted using a 50 mL centrifuge tube cut to 75 mm tall and 29 mm diameter.

### 2.4. Synthesis of ShEG Nanomaterials

For the high-shear exfoliation of EG, a Silverson L5M-A rotor stator mixer was used. The mixer head was lowered into the suspension, roughly 10 mm from the bottom of the vessel, and shear mixed for 30, 45, 60, and 75 min at 10,000 RPM to determine the optimum mixing time (ShEG-XX where XX denotes the time shear mixed). To prevent excessive heating and to improve exfoliation as reported by previous studies [26], the experiment was conducted with the vessel partially submerged in an ice water bath.

### 2.5. Synthesis of sEG Nanomaterials

For the probe sonication exfoliation of EG, a Fisher Scientific Model 705 sonic dismembrator was used with a 127 mm replaceable tip probe. The probe was lowered into the EG suspension in the same manner as the shear mixing procedure, roughly 10 mm from the bottom, and was sonicated at 50% amplitude for various times using a 3 s on and 3 s off pulse for total sonication times of 10, 15, 20, 25, and 30 min to determine the optimum sonication time (sEG-XX where XX denotes the period of time sonicated). The pulse was used to avoid excessive heating or evaporation of solvent along with submerging the vessel in an ice–water bath. The solution was also monitored by a temperature probe to keep the solution below 65 °C. The ShEG and sEG samples were freeze dried (Buchi Lyovapor L-200, Buchi Corporation, New Castle, DE, USA). The purpose of the freeze drying was to attempt to retain the separation of the graphene sheets [34,35].

### 2.6. Synthesis of Pd-Decorated Nanocomposites

Palladium (II) nitrate was added to 4 mg/mL of ShEG and sEG solutions with a target of a 20 wt.% Pd loading and stirred for 15 min. Sodium borohydride was then added in excess and stirred for an additional 30 min. The solution was centrifuged, rinsed 3 times and then left to dry in a 50 °C oven. As a control sample, EG that was not treated with the mechanical exfoliation was first bath sonicated to be dispersed in the same solvent ratio of 30% *v*/*v* ethanol/water for 25 min; then, it was subjected to the same procedure as previously described to prepare Pd-EG. Furthermore, Pd-NP was prepared in the same approach but without the addition of EG.

### 2.7. Structural Characterization

Scanning Electron Microscopy (SEM) (FEI Quanta FEG 250, FEI Company, Hillsboro, OR, United States) and Transmission Electron Microscopy (TEM) (FEI Titan 80-300 LB, FEI Company, Hillsboro, OR, USA) were used to investigate the morphology of the synthesized materials. Energy-dispersive X-ray spectroscopy (EDX) (Oxford XMax20, Oxford Instruments Group, Concord, MA, USA) was employed to investigate the elemental composition and distribution. X-ray photoelectron spectroscopy (XPS) (Scienta Omicron Inc., Colorado, MA, USA) was used to investigate the elemental composition as well as the valence states of the materials. C 1s, O 1s, Pd 3d, and Pd 3p high-resolution XPS spectra and survey spectra were obtained for all samples. The XPS data were analyzed using CasaXPS V2.3.23 software. All XPS spectra were corrected by the C 1s sp^3^ peak which was set to 285.0 eV. X-ray diffraction (XRD) crystallography was conducted on a PANanalytical Empyrean powder diffractometer with a Cu Κα (*λ* = 1.5405 Å) radiation source. Diffraction patterns were obtained using a spinner stage with a *2θ* range of 10 to 90°. XRD patterns were analyzed using X’Pert HighScore Plus V2.2.4 software. Raman spectroscopy (Renishaw InVia, Renishaw, Mississauga, ON, Canada) was conducted using a 532 nm laser from a range of 1200 to 2800 cm^−1^.

Further X-ray diffraction experiments were performed at the Canadian Light Source using the Brockhouse sector high-energy wiggler beamline [36]. All samples were measured at a temperature of 80 K using 55 keV X-rays. The total scattering patterns were collected using the Varex XRD 4343CT (Varex Imaging, Salt Lake City, UT, USA) flat-panel area detector. The detector was positioned at an inclined geometry bisected by the vertical scattering plane with a pitch angle of 30°. The detector was then translated such that the incident X-ray beam strikes the pixels along the bottom edge and 125° scattered X-rays impinge on the center-most pixel along the top edge. The scattering area used in the analysis was a wedge covering 90° in the azimuthal scattering angle *χ* and 40 Å^−1^ in *Q*. The collected total scattering patterns are simultaneously integrated to one-dimensional patterns in *Q* and corrected for measurement distortions using custom python code. The measurement distortions were quantified for the inclined geometry using a procedure outlined previously [37]. *G*(*r*) data analysis was performed using the PDFGUI [38].

### 2.8. Electrochemical Measurements

All electrochemical experiments were conducted using a CHI Potentiostat (CHI660E, CH Instruments, Inc., Bee Cave, TX, USA) with a three-electrode system. Cyclic voltammetry (CV) was conducted in a single chamber cell at 0.010 V/s, while linear sweep voltammetry (LSV) and chronoamperometry (CA) were conducted in a two-chamber U-cell with a cation exchange membrane separating the counter electrode from the working and reference electrode. A standard calomel electrode (SCE) (Hg/Hg_2_Cl_2_) filled with a 3 M KCl solution was used as a reference electrode, and a cleaned platinum wire was used as the counter electrode. To prepare the working electrode, 4 mg of the prepared materials was mixed with 100 μL Nafion binding agent (5 wt.%), 300 μL ethanol, and 600 μL pure water and sonicated for 20 min in a bath sonicator to disperse the mixture. Graphite foil was cut and pretreated by bath sonication for 10 min each in the sequence of pure water, 0.1 M HCl, pure water, and ethanol. Then, 70 μL of the nanomaterial ink was then drop cast on the pretreated carbon paper to have a geometric surface area of 1 cm^2^ and then left to dry overnight. Prior to each measurement, the electrolyte (0.5 M H_2_SO_4_) was purged with ultrapure argon for 20 min. The double-layer capacitance of the ShEG and sEG was calculated by CV using a potential window of 0.00 to 0.10 V at different scan rates varied from 0.010 to 0.100 V/s. The change in current at 0.05 V vs SCE was plotted against the scan rate, and the generated linear slope was used to calculate the double-layer capacitance.

## 3. Results and Discussion

### 3.1. Capacitance Studies of Mechanically Exfoliated EG

**Figure 1:**
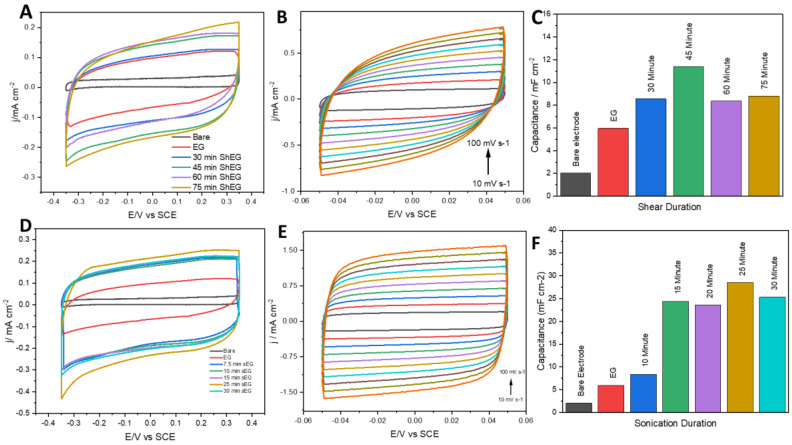
(**A**) Shear duration time study comparing the double-layer capacitance of bare graphite foil, EG, and EG after shearing for 30, 45, 60, and 75 min. (**B**) Cyclic voltammetry sweeping scan rate used to determine capacitance of ShEG after shear mixing for 45 min. (**C**) Summary of capacitances of the shear mixed materials. (**D**) Sonication duration time study comparing the double-layer capacitance of bare graphite foil, EG, and EG after high-powered tip sonication for 10, 15, 20, 25, and 30 min. (**E**) Cyclic voltammetry sweeping scan rate used to determine capacitance of ShEG after shear mixing for 45 min. (**F**) Summary of capacitances of the sonicated materials.

To determine the optimum period of processing time for the LPE procedure, the electrochemical double-layer capacitance in materials processed for different periods of time were compared. The samples which underwent high-shear for different times were compared first using CVs, as shown in Figure 1A. In Figure 1B, ShEG-45 is used as a representative sample in CV and shows sweeps at varying scan rates from 0.010 to 0.100 V/s where the difference in current density between the oxidative and reductive sweeps at 0 V was taken and is used to determine the capacitance of each material. It can be observed that 45 min has a higher capacitance than the other shear exfoliation times, as seen in Figure 1C, and thus was used for further experiments. The same experiments were conducted for the probe sonication exfoliation method. The CV curves shown in Figure 1D demonstrate that sEG-25 has the highest double-layer capacitance. Representative CV curves are shown in Figure 1E for the determination of the capacitance in the same manner as previously described. To confirm the highest capacitance, the same calculation as the ShEG materials was conducted and is shown in Figure 1F. Capacitance is commonly used to compare the performance of graphene. Additionally, the shape of the curve can also be used to describe the electrochemical behavior of the material, where a more rectangular CV curve implies higher capacitive performance [39]. The comparison in Figure 1B,E shows that sonication has a more rectangular CV shape. The electrochemically active surface area (ECSA) is associated with the capacitance. As shown in Table S1, sEG-25 exhibits the highest ECSA.

### 3.2. Morphological Characterization of Graphene-Based Nanomaterials

**Figure 2:**
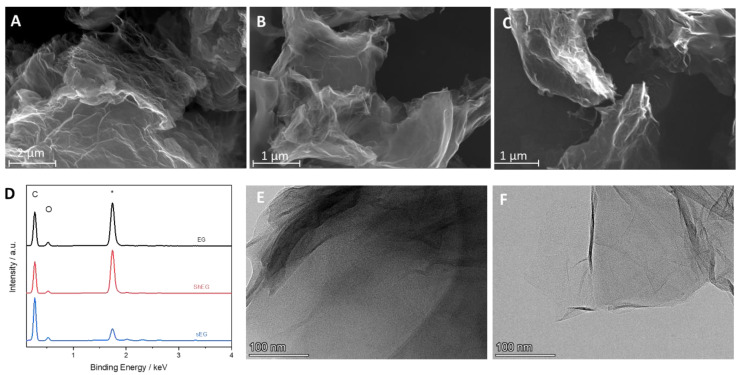
SEM images of (**A**) EG, optimized (**B**) ShEG, (**C**) sEG samples. (**D**) EDX of the prepared graphene-based nanomaterials where the carbon and oxygen peaks are labeled and the substrate silicon peak labelled with *. TEM images of optimized (**E**) ShEG and (**F**) sEG samples.

SEM and TEM were used to investigate the morphology of the synthesized nanomaterials and nanocomposites. Representative images of EG, ShEG, sEG are shown in Figure 2. The starting material EG in Figure 2A had a porous sponge-like structure which is comparable to untreated graphite flakes but with higher interlayer distances. The increase in interlayer distance between the carbon sheets was a result of the oxidative chemical procedure and the intercalation of ions followed by thermal treatment, which caused an expansion and mild exfoliation of graphite. The mechanical LPE process was successful in producing characteristic crumpled graphene sheets, as seen in Figure 2B,C. Figure 2D displays the EDX spectra of the samples, where the presence of C and O was confirmed for the EG, sEG and ShEG samples. The atomic percentages of C and O for each sample were calculated and are listed in Table S2. It is confirmed that the mechanical exfoliation procedures do not introduce oxygen content to the nanomaterials. Further TEM images of ShEG and sEG are shown in Figure 2E,F, respectively, where characteristic graphene wrinkled sheets were observed. The ShEG showed a lower degree of exfoliation with darker areas representing a thicker stack of interconnected graphene sheets while sEG exhibited a lighter and more exfoliated material.

**Figure 3:**
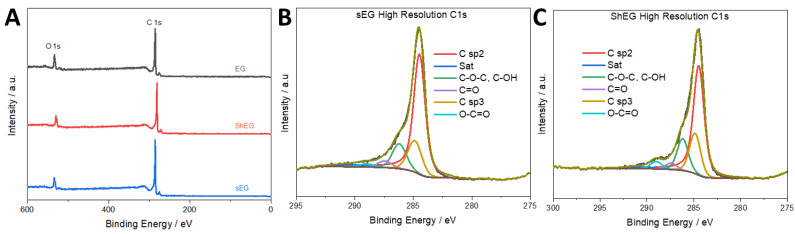
(**A**) XPS survey scan of the synthesized nanomaterials. (**B**) XPS high-resolution C1s scan of the prepared sEG nanomaterial and (**C**) ShEG.

Further elemental analysis in XPS also confirmed the presence of C and O in the nanomaterials. Figure 3A survey scans reveal that each sample has a C 1s peak at roughly 284 eV and an O 1s peak at roughly 532 eV. In Figure 3B,C, high-resolution scans of the C 1s peak are shown for the optimized sEG and ShEG nanomaterials. Peaks at roughly 284, 286, 287, 289, and 290 eV are attributed to C=C/C-C, C-O-C/C-OH, C=O, O-C=O, and π-π*, respectively. Similar results were seen in Figure S3B. The primary peak observed is the C=C peak, which is characteristic of graphitic materials.

**Figure 4:**
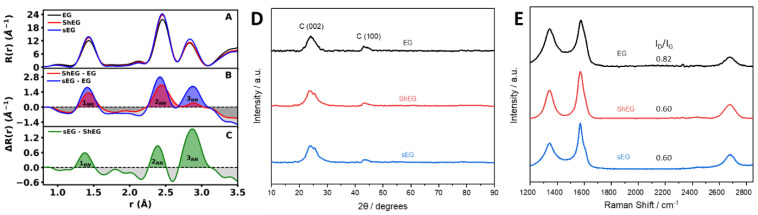
(**A**) Experimental *R*(*r*) of the substrates EG, optimized ShEG and sEG samples. (**B**) Experimental Δ*R*(*r*) of ShEG and sEG by subtraction of EG substrate. (**C**) Experimental Δ*R*(*r*) of sEG by subtraction of ShEG substrate. The increased intensity for the characteristic graphene C-C basal-plane nearest-neighbor (NN) distances are shaded in red, blue and green while the reduced functional group intensities are shaded in gray. (**D**) XRD patterns of nanomaterials prepared through mechanical exfoliation using shear and sonication. (**E**) Raman spectroscopy of the various prepared nanomaterials with the defect density, *I_D_/I_G_*, listed for each nanomaterial.

Radial distribution function *R*(*r*) analysis of the substrates can provide unique information about the degree of defects and quantity of functional groups which conduct the dispersion and size of the nanoparticles grown in situ within the substratum. In Figure 4A, the *R*(*r*) for all substrates is shown. Each *R*(*r*) shows a similar pattern with slight variations in intensity. Taking the differential radial distribution functions Δ*R*(*r*) between substrates, more information is gleaned about the changes that each of the LPE techniques introduce into the substrate. In Figure 4B, the Δ*R*(*r*) was calculated between the LPE substrates (ShEG, sEG) and the untreated EG. The LPE substrates show increased and sharper correlations in the characteristic graphene C-C basal-plane nearest-neighbor (NN) distances of 1.43, 2.45 and 2.84 Å. These changes are in conjunction with a decrease in intensity around the respective peaks. The correlations around the characteristic graphene NN distances are a result of functional groups such as O-H, C-H, C-O and C-C defects from pristine graphene locations. In concert, these changes indicate a reduction in the basal–plane defects and functional groups for both LPE substrates, as compared to EG. Furthermore, comparing the LPE substrates to one another, ShEG shows an increase in only the 1st and 2nd nearest-neighbor distances at 1.43 and 2.45 Å, respectively. This indicates that defects are reduced in degree but are not completely removed due to the missing 3rd nearest-neighbor distance at 2.84 Å. Defects which could explain the missing intensity of the 3rd NN include intrinsic graphene defects such as Stone–Wales, single and multiple vacancy. Conversely, sEG produces a similar trend; however, additional intensity is observed at the 3rd NN distance, indicating a mix of partial and complete reduction in defects occurred as compared to ShEG. The changes between ShEG and sEG substrates are further highlighted in Figure 4C.

Further investigation of the nanomaterials was conducted with XRD measurements to probe the structural and crystallite characteristics. Each pattern shows a characteristic C (002) plane peak at roughly *2θ* = 25° in Figure 4D. The samples presented show evidence of successful exfoliation of the graphite sheets into graphene sheets from the broadening of the C (002) peak.

Through Raman spectroscopy, the relative number of defects in the samples can also be investigated. By taking the intensity of the D-band (*I_D_*) at roughly 1350 cm^−1^ and dividing the intensity of the G-band (*I_G_*) at roughly 1600 cm^−1^ as seen in Figure 4E, the *I_D_/I_G_* ratio is obtained, where higher ratios imply an increased number of defects in the graphene sheet. A third peak is also present at roughly 2700 cm^−1^ known as the 2D-band, which is an overtone of the D-band. The G-band relates to the in-plane stretching of the graphene sheet, while the D-band relates to the in-phase vibrations of the hexagonal ring of carbon atoms in a radial direction [40]. The change in *I_D_/I_G_* from the starting material, EG, to the mechanically exfoliated samples demonstrates that the process did not introduce any additional defects but produces graphene with reduced defects. This trend agrees well with the reduction in graphene defects observed in the *R*(*r*) analysis of the substrates.

### 3.3. Characterization of Palladium Nanoparticle Functionalized Nanocomposites

**Figure 5:**
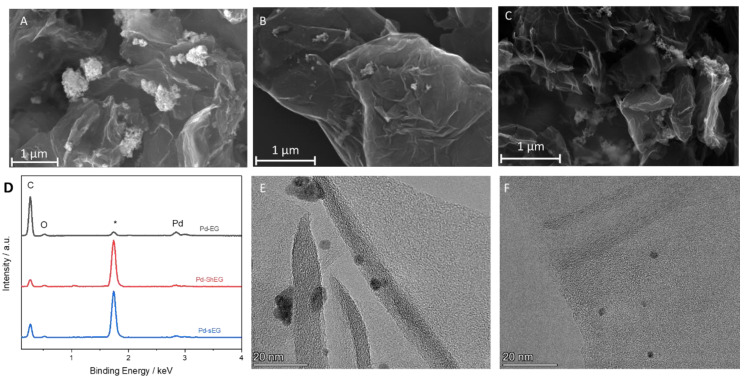
SEM images of (**A**) Pd-EG (**B**) Pd-ShEG (**C**) Pd-sEG nanocomposites. (**D**) EDX spectra of Pd-functionalized nanocomposites with the carbon, oxygen, and palladium peaks are labelled and the substrate silicon peak labelled with *.TEM images of (**E**) Pd-ShEG and (**F**) Pd-sEG nanocomposites.

Similar to the non-functionalized nanocomposites, the Pd-NP functionalized nanocomposites were investigated through SEM, EDX, and TEM. The decoration of Pd on EG did not change the morphology but had a notably larger sized Pd-NP on the edges of the EG structures as seen in Figure 5A. In contrast, small Pd particles were seen for both Pd-ShEG (Figure 5B) and Pd-sEG (Figure 5C), showing that the higher degree of exfoliation produced by the sonication was favorable for the uniform distribution of the Pd-NP. EDX was also used to investigate the elemental composition of the prepared nanocomposites in Figure 5D. The presence of C, O, and Pd was confirmed for all materials with similar compositions as listed in Table S1 and had a consistent Pd-NP atomic percent of roughly 2%. In the case of EDX mapping (Figure S1), the uniform distribution of Pd on the sEG surface was observed. Further imaging using TEM was conducted, showing finely exfoliated graphene with Pd-NP functionalized on the sheet. Further analysis allowed for the determination of particle sizes, where smaller Pd-NP sizes in Pd-sEG (~3.8 nm) were found compared to Pd-ShEG (~7.0 nm).

**Figure 6:**
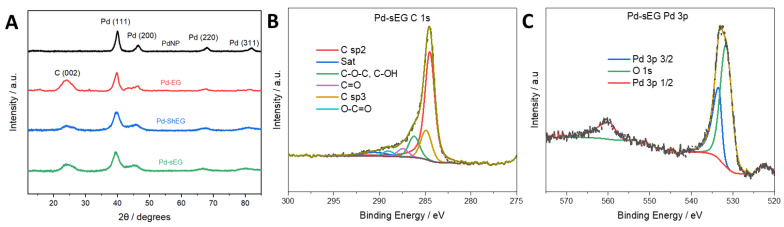
(**A**) XRD patterns of Pd-functionalized nanomaterials with peaks attributed to the Pd (111), Pd (200), Pd (220), and Pd (311) planes. (**B**) XPS high-resolution scan of Pd-sEG C 1s. (**C**). XPS high-resolution scan of the Pd 3p and O 1s peaks of Pd-sEG nanocomposite.

XRD analysis was also conducted on the nanocomposites. In Figure 6A, Pd-NP is shown and compared to the Pd-NP functionalized nanocomposites. In each pattern, the peaks present are at roughly 40, 45, 67, and 80°, which are attributed to the Pd (111), Pd (200), Pd (220), and Pd (311) planes, respectively, with Pd (111) being the dominant plane observed. The characteristic C (002) peak is still observed at *θ* = 25 °. As the FWHM of the C (002) peak was consistent with non-functionalized samples, the functionalization process did not affect the degree of exfoliation. To confirm EDX results, XPS was also conducted on the nanocomposites. Using the material with the highest activity, Pd-sEG, as an example, the high-resolution scan of C 1s (Figure 6B) shows that the carbon structure is similar to non-functionalized materials, as seen in Figure S3B. Furthermore, a high-resolution XPS scan of Pd 3p (Figure 6C) confirms the presence of Pd metal. In the survey scan (Figure S2), the Pd-NP-decorated samples also contain Pd 3d peaks at roughly 340 and 344 eV, which imply the presence of metallic Pd, as seen in Figure S3A in high-resolution scans of the Pd 3d peaks. Furthermore, for these samples, the Pd 3p_3/2_ peak is deconvoluted from the O 1s intensity using the Pd 3p_1/2_ peak, which results due to spin orbit coupling. The Pd 3p_1/2_ peak intensity is known to have a 1:2 ratio to the Pd 3p_3/2_ such that the remaining area of the convoluted peak can be attributed to oxygen intensity [41], as seen in Figure 6C.

Nanoparticle size determination is conducted through Bragg diffraction, TEM images, and *G*(*r*) analysis. Specifically, through modeling of the *G*(*r*), accurate mean spherical nanoparticle diameters can be determined for each sample. Methods such as TEM images and Bragg diffraction can often under-represent smaller-sized particles [42]. For accurate size determination, the instrument broadening factors are first determined through *G*(*r*) modeling of a Sigma-Aldrich powdered diamond sample. The palladium mean diameter was refined to values of 8.4 ± 0.13, 5.6 ± 0.12, 4.2 ± 0.11 and 3.5 ± 0.09 nm for nanoparticles deposited in ex-substratum, EG, ShEG and sEG substrates, respectively. This trend in nanoparticle size is observed between all methodologies and is listed in Table S2. Additionally, the trend follows that of the defect and functional group concentration observed within the substrates from Raman and *R*(*r*) analysis. As the defect density decreases and functional groups are reduced, there are fewer binding sites available, resulting in smaller, more well-dispersed nanoparticles [32].

**Figure 7:**
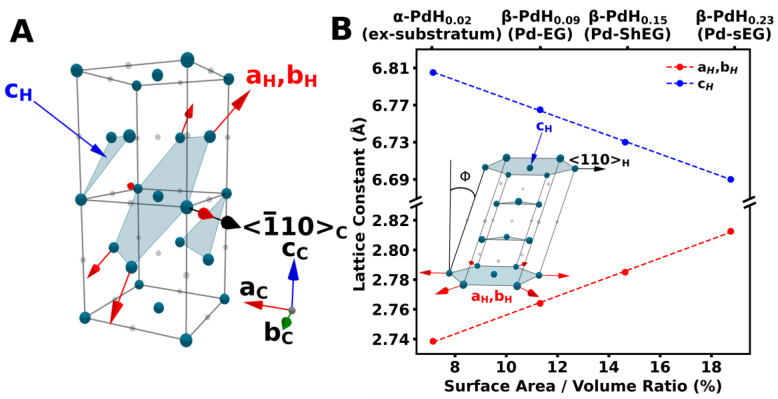
(**A**) Diagram highlighting the choice between cubic and hexagonal conventions for the face-centered cubic PdH_x_ unit cell. (**B**) Comparison of the pseudo-hexagonal lattice constants and the surface area to volume ratio. Inset shows the pseudo-hexagonal unit cell convention. Blue arrows represent the compression along the *c_H_* axis, red arrows represent expansion along *a_H_, b_H_* axes and black is the strain of the cubic {111}_c_ plane in the <$\bar{1}10$>_c_ or equivalently <110>_H_ direction. Additionally, the tilt angle Φ is shown.

Pd-NP has a high affinity for hydrogen sorption, making them an ideal additive to EG for solid-state hydrogen storage. Total scattering studies of Pd-NP using pair *G*(*r*) and radial *R*(*r*) distribution function analysis can provide unique information about the structural characterization, deformation, and composition of these nanoparticles. Total scattering studies were performed for all palladium nanoparticles grown in situ for all investigated LPE methodologies. Additionally, total scattering studies were performed for all substrates prepared using all LPE methodologies. Through subtraction of the substrate signal from the respective Pd deposited substrates, the total scattering from only the Pd nanoparticles is isolated. We observed the formation of core–shell PdH_x_ nanoparticles in all cases. The core component is found to be nanocrystalline, while conversely, the shell component is disordered. PdH_x_ has previously been shown to form core–shell structures [43,44,45]. Commonly, the nanocrystalline component is reported as face-centered cubic (FCC) PdH_x_. Here, both core and shell components are fit to a strained FCC PdH_x_ phase. The strain occurs along the <$\bar{1}10$>_c_ slip direction for the cubic {111}_c_ slip plane. Similarly strained pseudo-cubic systems have been observed through X-ray and neutron powder diffraction [46,47,48,49]. To simplify the unit cell distortion caused by the strain, a hexagonal convention of FCC can be chosen. The strain along the <110>_H_ direction is then accounted for by producing a pseudo-hexagonal unit cell with an *α*, *β* angle of 89.25° for the core and 81.65° for the shell, which produces a tilt angle Φ  of 1.5° and 16.7°, respectively. The tilt angle is found to be constant for all nanoparticle sizes. The conversion from cubic to hexagonal convention used is shown in Figure 7A. Expansion of the *a_H_* and *b_H_* axes and the compression of the c_H_ axis are found to be proportional to the surface area to volume ratio. Conversely, the lattice constants of the shell phase were found to be constant for all nanoparticle sizes. The comparison of the core component lattice constants to the surface area to volume ratio is shown in Figure 7B.

**Figure 8:**
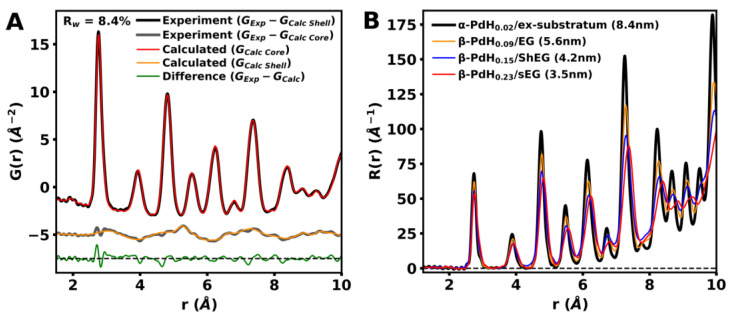
(**A**) Experimental *G*(*r*) of the remaining Pd structures after subtraction of the sEG substrate. Experimental *G*(*r*) is fit to the theoretical core–shell model using a β-PdH_0.23_ pseudo-hexagonal core and a disordered PdH_x_ pseudo-hexagonal outer shell. (**B**) Experimental *R*(*r*) of PdH_x_ core components grown in situ on ex-substratum, EG, ShEG and sEG substrates. The *R*(*r*) highlights the distortion which is occurring from the ideal PdH_x_ FCC unit cell with decreasing nanoparticle size and increased hydrogen content.

Using the pseudo-hexagonal unit cell, the experimentally measured *G*(*r*) for palladium nanostructures grown in situ in each substrate were modeled. The calculated *G*(*r*) core–shell model for β-PdH_0.23_/sEG is shown in Figure 8A. The phase fractions of the core and shell components were found to be proportional to thesurface area to volume ratio as expected for a core–shell system. The phase fractions are shown in Figure S4 in the supplemental materials. The core and shell components are separated, and the total model error is split between each component by a phase fraction.

The appearance of a small peak in the *G*(*r*) as observed in Figure S5C,D for Pd-ShEG and Pd-sEG at 2 Å confirms the presence of hydrogen within the system, and that hydrogen content is increasing as the nanoparticle size decreases. The hydrogen content of the PdH_x_ is often calculated as a function of the cubic lattice constant b [50,51,52]. Here, the system is pseudo-cubic; to account for this, the relationship provided [51] is recalculated as a function of pseudo-hexagonal unit cell volume, as shown in Figure S6A. Using this relationship, the hydrogen content for each group of palladium nanoparticles is calculated. The calculated hydrogen content is found to be proportional to the surface area to volume ratio, as observed in Figure S6B. The remaining experimental *G*(*r*) values are presented in the supplementary materials in Figure S7. The aforementioned HRPDF results were consistent with the hydrogen uptake measured by the electrochemical technique.

### 3.4. Hydrogen Uptake and Release Performance of Pd-EG Nanomaterials

**Figure 9:**
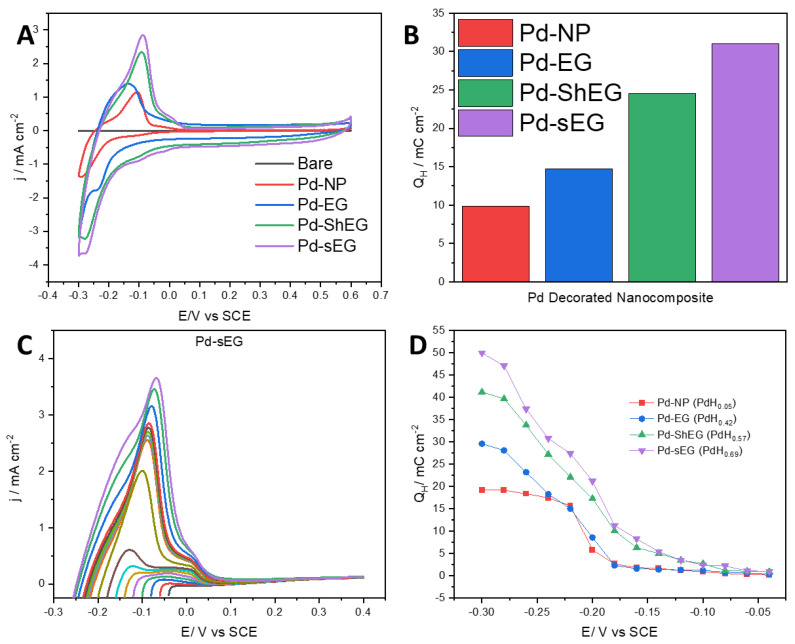
(**A**) Cyclic voltammetry of the various nanomaterials prepared without mechanical exfoliation, with sonication and shear, as well as Pd-NP, EG, and bare carbon paper. (**B**) The associated integration of the curve for the desorption charge for nanomaterials with Pd deposition. (**C**) LSV after holding the potential at −0.300 V for 5 min and varying the starting potential from −0.300 to −0.040 V. (**D**) Overall hydrogen desorption charge, *Q_H_*, obtained from integration of LSVs of the various nanomaterials including Pd-NP, Pd-EG, Pd-ShEG, and Pd-sEG.

To test the material’s hydrogen uptake and release ability, CV curves of the palladium-decorated nanomaterials were conducted in the same conditions as previously stated. Figure 9A compares Pd-NP vs. the prepared nanomaterials from a potential range of −0.3 to 0.6 V vs. SCE with two clear peaks. The peaks are interpreted as the adsorption of hydrogen from roughly 0 to −0.3 V and desorption of hydrogen from roughly −0.3 to 0.05 V. The material with the highest adsorption and desorption is Pd-sEG, which demonstrates that sonification exfoliation has the ability to improve hydrogen storage. Similarly, Pd-ShEG, high shear mixing exfoliation is also shown to improve the adsorption and desorption of hydrogen over the base Pd-EG material. Moreover, Pd-NP with their electrochemical activity normalized by Pd wt.% demonstrates that a synergistic effect occurred when they were combined with EG in Figure 9A. The mechanism for the hydrogen uptake and release can be derived based on the Volmer process [53]. The process can therefore be explained through the scheme $M+e^{-}+H^{+}\;⇌H_{ad}-M$, where M represents the material or sample. Further analysis was conducted by taking the integration of the anodic desorption peak to calculate the hydrogen desorption charge, *Q_H_*, which gives a measure of how much hydrogen is released after uptake. Figure 9B confirms that Pd-sEG has the highest hydrogen desorption charge (31.05 mC cm^−2^) as compared to Pd-ShEG (24.54 mC cm^−2^), Pd-EG (14.70 mC cm^−2^) and Pd-NP (9.87 mC cm^−2^). This demonstrates again that sonication is effective in improving the hydrogen storage ability. The improvement might be explained by the effective exfoliation caused by the sonication and high shear mixing process and increase in surface area allowing for a higher availability of Pd-NP for hydrogen uptake and release.

Further electrochemical testing was conducted using a U-cell with the working electrode separated from the Pt wire counter electrode with a cation exchange membrane. The use of a U-cell to separate the working electrode from the counter electrode is to address concerns relating to the deposition of Pt on the working electrode. Pt deposition is more likely to occur in experiments with held potentials and repeated cycling [54]. As a result, this effect was only considered for the following analysis. First, the time required to achieve maximum hydrogen storage capacity was determined using LSV and CA. CA was conducted by holding the potential at −0.3 V for 1, 3, 5, 7 and 10 min. After each hold time, an LSV was conducted sweeping from −0.3 to 0.6 V at a 0.010 V/s scan rate. It was seen that the current density was increased to a maximum. To determine the optimum holding time, the integration of the anodic sweep was calculated for each curve for each nanocomposite and compared against the hold time. It is seen that across all materials, there is a plateau of *Q_H_* after 3 min, indicating a complete saturation of hydrogen. To ensure a maximized hydrogen sorption, CA was conducted for 5 min for all further experiments. In a different set of experiments, after a 5-min CA hold was conducted at different potentials, LSVs were immediately run at the held potentials from −0.3 to −0.04 V to a consistent 0.4 V upper potential. The set of LSV scans for Pd-sEG is shown as a representative graph in Figure 9C. For each material, the *Q_H_* was determined for each different held potential shown in Figure 9D.

For the scans shown, a major desorption peak is seen with a shoulder peak present. The two desorption peaks can be attributed to different phases of Pd-hydride, which are dependent on the applied potential. The α-phase hydride is associated with hydrogen sorption at lower concentrations as a solid solution, while the β-phase hydride relates to higher concentrations of hydrogen in the material as a hydride. Furthermore, an intermediate α-β or α’ phase exists between the α and β phases [19,55,56]. It is shown that over the range of the different potential sweeps, Pd-sEG had the highest *Q_H_* (50.40 mC cm^−2^), while Pd-ShEG showed slightly lower activity (41.20 mC cm^−2^), and both LPE samples had notably improved activity over the base materials (29.60 mC cm^−2^ for Pd-EG and 19.15 mC cm^−2^ for Pd-NP). From Figure 9D, insight into the phases of Pd-hydride can also be obtained. The slight increase in *Q_H_* in the potential range from −0.040 to −0.190 V is attributed to increased capacity for α-phase hydride, while the range from roughly −0.200 to −0.260 V would be the α-β phase hydride transition phase. Finally, the higher potential range of roughly −0.280 to −0.300 V can be attributed to β-hydride formation. For Pd-NP and Pd-EG, it is also seen that a plateau of *Q_H_* is seen from −0.220 to −0.300 V, which implies a maximized hydrogen sorption, while there is continued increase for the LPE samples. This increase in hydrogen sorption could be attributed to the increased hydrogen spillover caused by the higher exfoliation as evidenced in SEM and XRD as well as higher capacitance. In previous studies, it has been reported that the transition between the α-β phase hydride affects the hydrogen sorption as the rate-determining step [57]. It can therefore be inferred that a higher α-β phase also implies a higher hydrogen uptake. The PdH_x_ was also calculated and listed in Figure 9D based on *Q_H_*.

It was previously confirmed in Table S1 that the exfoliation methods increased the surface area of materials. The increase in surface area can therefore be correlated to the hydrogen uptake and release ability of materials. It has been seen that samples that have increased surface area allow for a more uniform distribution of Pd-NP and higher Q_H_. This could be due to a higher number of active sites allowing for more effective hydrogen spillover effect through a more effective diffusion of H [33].

Stability testing was also conducted on the Pd-functionalized nanocomposites through repeated cycling of cyclic voltammetry. The experiments were conducted at a 0.020 V/s scan rate with a potential window of −0.3 to 0.6 V. As previously stated, due to concerns regarding the deposition of Pt from the counter electrode, the experiments were conducted using a U-cell with a cation exchange membrane separating the working and reference electrode from a Pt wire counter electrode. The electrolyte utilized was the same as previous experiments: 0.5 M H_2_SO_4_ initially purged with Ar gas. The cycling stability tests demonstrated there is a only slight decrease in activity, as shown in Figure S8.

## 4. Conclusions

To conclude, the use of mechanical liquid phase exfoliation of expanded graphite increases the surface area to allow for a more dispersed decoration of Pd-NP compared to the base material. The increased surface area and higher dispersed Pd-NP allows for more accessibility for H-sorption due to a higher number of active sites. This is confirmed based on morphological and electrochemical characterization demonstrating the efficacy of the utilized high-shear mixing exfoliation and probe-tip sonication as pretreatments. The optimized processing time for both high-shear mixing and probe-tip sonication was determined through the use of capacitance studies. After the mechanical exfoliation of graphite to graphene, the use of freeze drying was used to prevent the agglomeration of the carbon sheets prior to the decoration with Pd-NP. The hydrogen storage uptake and release of Pd-sEG was the highest when compared to the base materials of Pd-EG and Pd-NP, while Pd-ShEG also improved the capacity. The advanced HRPDF analysis confirmed the formation of PdH_x_ and provided insights in the enhancement of hydrogen storage of Pd-sEG and Pd-ShEG. The strong linear relationship with effective surface area to volume ratio revealed nanoparticle size as a determining factor for the hydrogen uptake and release. Ultimately, the investigation in this work of using LPE over traditional chemical methods to improve solid-state hydrogen storage took a notable step away from fossil fuels toward a hydrogen economy using safer synthesis methods. Future work could include the use of heteroatom doping, which could further improve the hydrogen uptake/release of mechanically exfoliated graphene nanomaterials.

## Data Availability

The data presented in this study are available on request from the corresponding author.

## Supplementary Materials

The following supporting information can be downloaded at: https://www.mdpi.com/article/10.3390/nano13182588/s1, Figure S1: EDX element maps of Pd-sEG showing the distribution of carbon, oxygen, and palladium on the nanocomposite. Figure S2: XPS survey scans of the Pd-functionalized nanomaterials. Figure S3: (A) High-resolution XPS scans of Pd 3d of each palladium-decorated nanomaterial and (B) high-resolution XPS scans of each nanomaterial and functionalized nanocomposite. Figure S4: Comparison of the PdHx nanoparticle core–shell phase fractions and the particle surface area to volume ratio. Both core and shell phase fractions are found to be proportional to the nanoparticle surface area volume ratios. Figure S5: Experimental *G*(*r*)’s of the remaining Pd structures after subtraction of the substrates (a) ex-substratum (b) EG, (c) ShEG and (d) sEG. The experimental *G*(*r*) values are fit to a theoretical core–shell model using a nanocrystalline PdHx pseudo-hexagonal core and a disordered PdHx pseudo-hexagonal outer shell. The core and shell components are separated, and the total model error is split between each component by phase concentration. Figure S6: (A) Comparison of PdHx H/Pd ratio and the hexagonal unit cell volume. The H/Pd ratios are given as a function of cubic lattice constant. Under the hexagonal convention, the H/Pd ratios can be replotted against unit cell volume. Using this new relationship, the H/Pd ratio of the palladium nanoparticles in this experiment can be calculated. (B) Comparison of the calculated PdHx H/Pd ratio and the surface area to volume ratio. The calculated H/Pd ratio is found to be proportional to the surface area to volume ratio. Figure S7: Experimental G(r) values of the remaining Pd structures after subtraction of the substrates: (a) ex-substratum, (b) EG, (c) ShEG and (d) sEG. The experimental G(r) values are fit to the theoretical core–shell model using a nanocrystalline PdHx pseudo-hexagonal core and a disordered PdHx pseudo-hexagonal outer shell. The mean spherical diameter is refined to 8.4 ± 0.13, 5.6 ± 0.12, 4.2 ± 0.11, 3.5 ± 0.09 nm for ex-substratum, EG, ShEG and sEG, respectively. Figure S8: Stability testing of Pd-functionalized nanocomposites (A) Pd-ShEG and (B) Pd-sEG conducted through repeated CV cycling. Table S1: Capacitances of the graphene-based nanomaterials. Table S2: EDX results showing the atomic percent of carbon, oxygen, and palladium of each material and nanocomposite. Table S3: PdHx nanoparticle pseudo-hexagonal core–shell model parameters.
