# Supplementary material for: Mechanical Exfoliation of Expanded Graphite to Graphene-Based Materials and Modification with Palladium Nanoparticles for Hydrogen Storage
Source: Nanomaterials (Basel). 2023 Sep 19;13(18):2588. doi: 10.3390/nano13182588 (PMC10534434; doi:10.3390/nano13182588)
Supplement: Supplementary file 1 [file nanomaterials-13-02588-s001.zip › nanomaterials-2593629-supplementary.pdf]

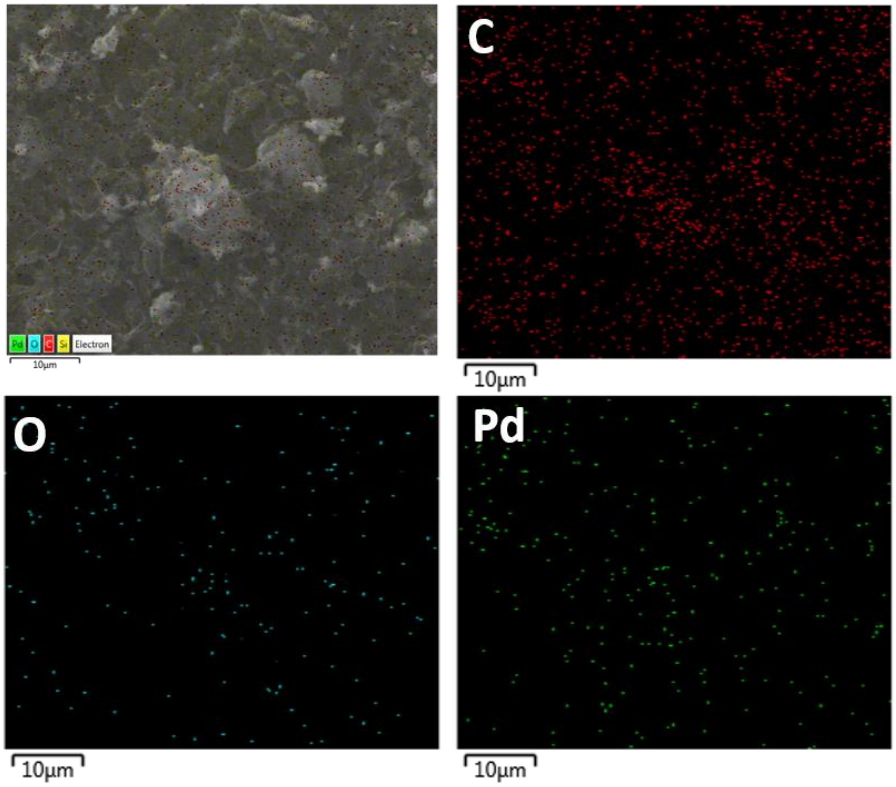

Figure S1: EDX element maps of Pd-sEG showing the distribution of carbon, oxygen, and palladium on the nanocomposite.

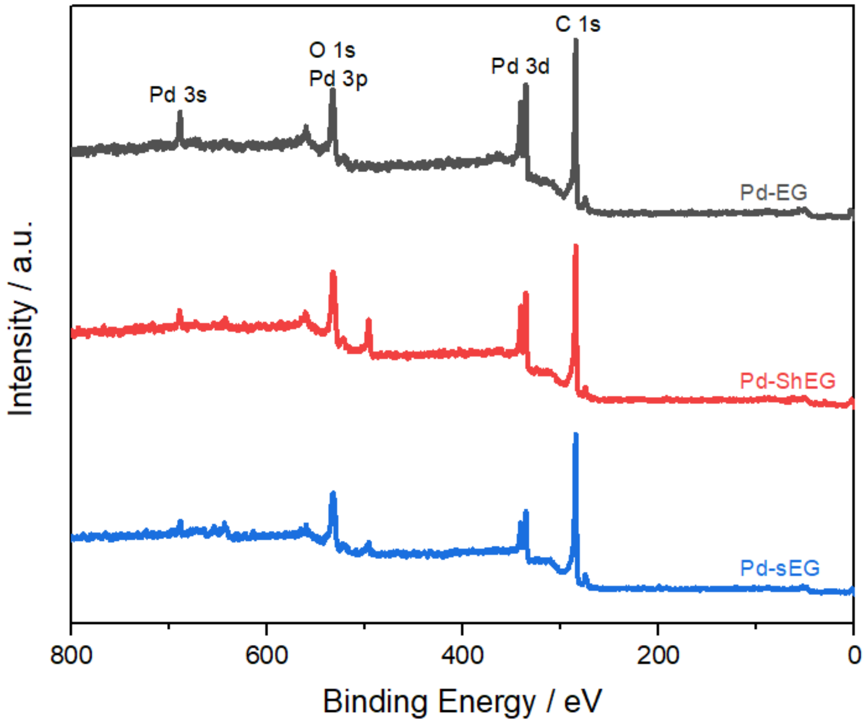

Figure S2: XPS survey scans of the Pd functionalized nanocomposites.

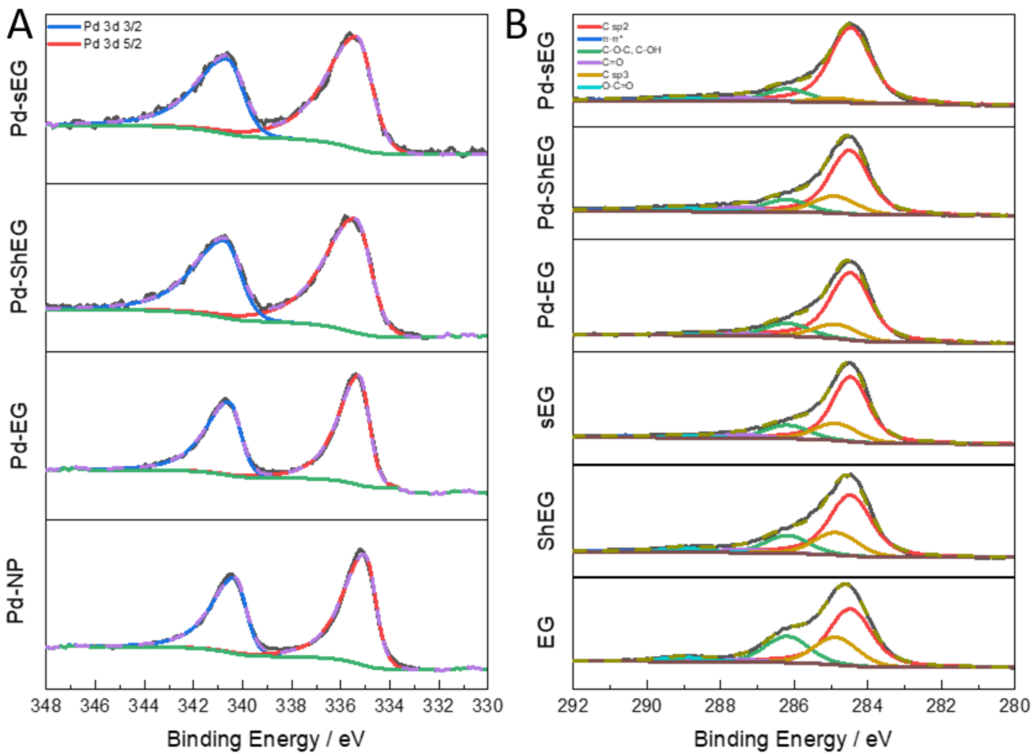

Figure S3: A) High resolution XPS scans of Pd 3d of each palladium decorated nanomaterial and B) high resolution XPS scans of each nanomaterial and functionalized nanocomposite.

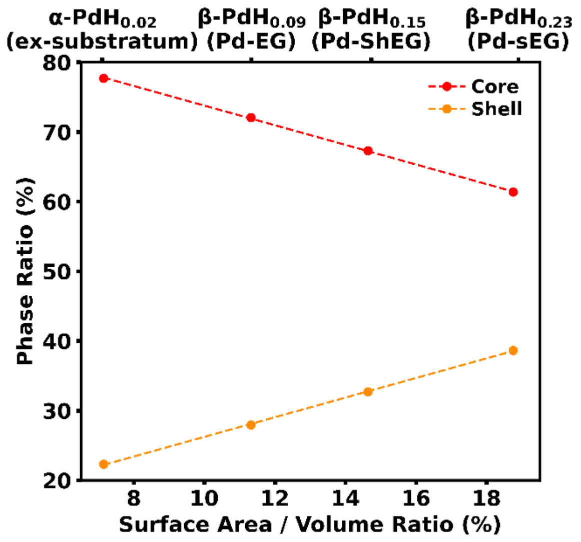

Figure S4: Comparison of the PdH_x nanoparticle core-shell phase fractions and the particle surface area to volume ratio. Both core and shell phase fractions are found to be proportional to the nanoparticle surface area to volume ratios.

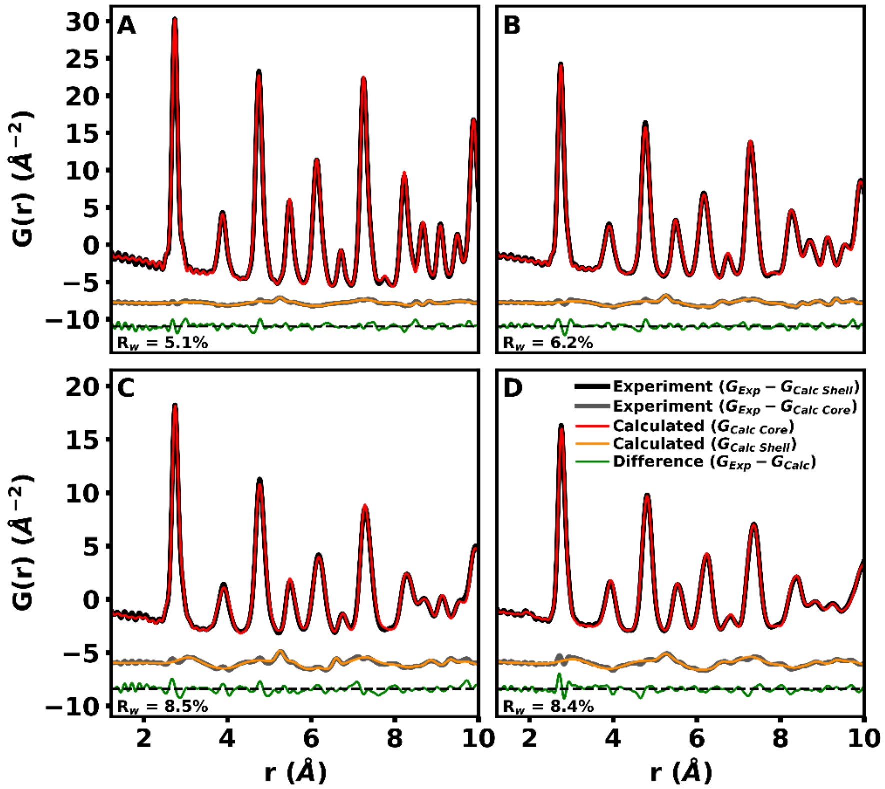

Figure S5: Experimental *G(r)*'s of the remaining Pd structures after subtraction of the substrates A) ex-substratum, B) EG, C) ShEG and D) sEG. The experimental *G(r)*'s are fit to a theoretical core-shell model using a nanocrystalline PdH_x pseudo-hexagonal core and a disordered PdH_x pseudo-hexagonal outer shell. The core and shell components are separated, and the total model error is split between each component by phase concentration.

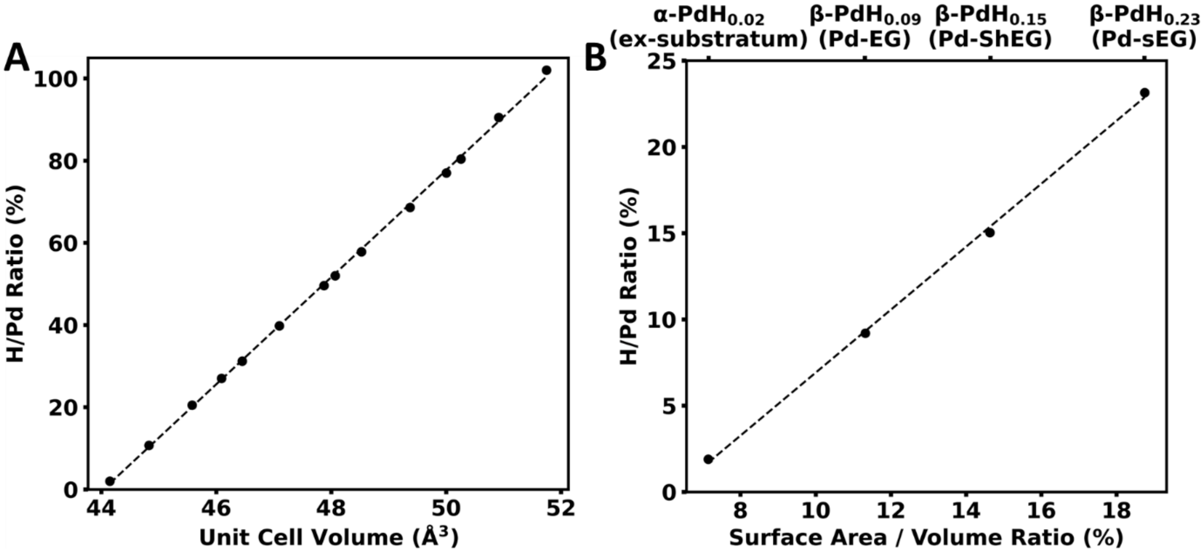

Figure S6: A) Comparison of PdHx H/Pd ratio and the hexagonal unit cell volume. The H/Pd ratios are given as a function of cubic lattice constant. Under the hexagonal convention, the H/Pd ratios can be replotted against unit cell volume. Using this new relationship, the H/Pd ratio of the palladium nanoparticles in this experiment can be calculated [1]. B) Comparison of the calculated PdHx H/Pd ratio and the surface area to volume ratio. The calculated H/Pd ratio is found to be proportional to the surface area to volume ratio.

[1] Hijazi, I.; Zhang, Y.; Fuller, R. A Simple Palladium Hydride Embedded Atom Method Potential for Hydrogen Energy Applications. *Journal of Energy Resources Technology, Transactions of the ASME* **2019**, *141*, doi:10.1115/1.4042405.

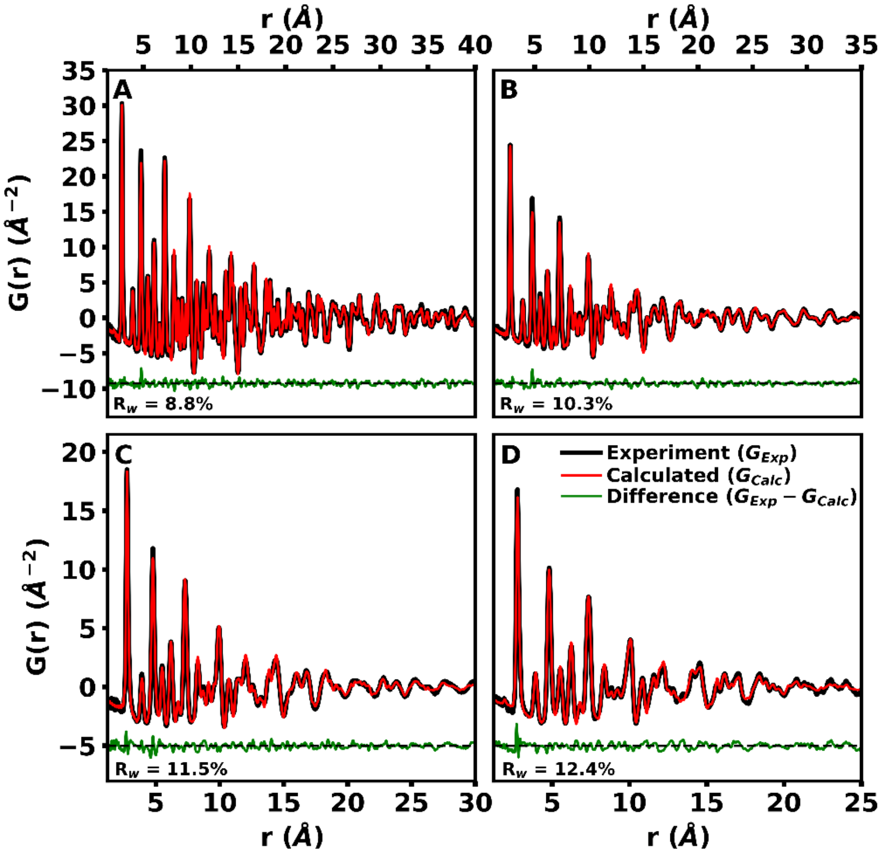

Figure S7: Experimental *G(r)*'s of the remaining Pd structures after subtraction of the substrates A) ex-substratum, B) EG, C) ShEG and D) sEG. The experimental G(r)'s are fit to the theoretical core-shell model using a nanocrystalline PdH_x pseudo-hexagonal core and a disordered PdH_x pseudo-hexagonal outer shell. The mean spherical diameter is refined to 8.4 ± 0.13, 5.6 ± 0.12, 4.2 ± 0.11, 3.5 ± 0.09 nm for ex-substratum, EG, ShEG and sEG, respectively.

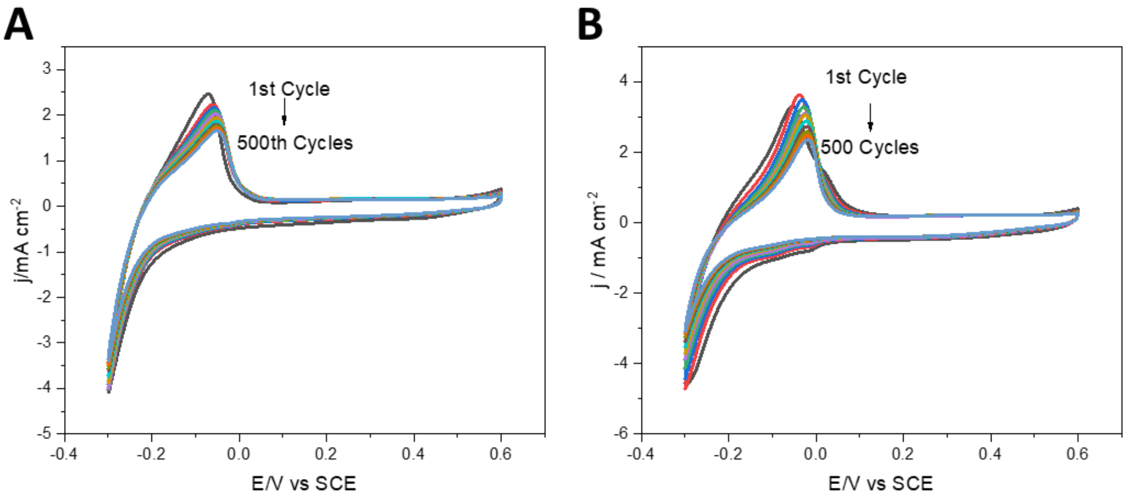

Figure S8: Stability testing of Pd functionalized nanocomposites A) Pd-ShEG and B) Pd-sEG conducted through repeated CV cycling.

Table S1: Capacitances of the graphene-based nanomaterials.

Sample	Bare Electrode	EG	ShEG -30	ShEG -45	ShEG -60	ShEG -75	sEG-10	sEG -15	sEG -20	sEG -25	sEG -30
Capacitance (mF cm^{-2})	2.02	5.97	8.55	11.35	8.37	8.81	23.11	24.4	23.6	28.5	25.3

Table S2: EDX results showing the atomic percent of carbon, oxygen, and palladium of each material and nanocomposite.

Material	Carbon	Oxygen	Palladium
EG	81.8	18.2	0.0
sEG	85.2	14.8	0.0
ShEG	84.8	15.2	0.0
Pd-EG	78.4	15.4	1.7
Pd-sEG	81.6	16.4	2.0
Pd-ShEG	79.1	18.9	2.1

Table S3: PdH_x nanoparticle pseudo-hexagonal core-shell model parameters.

Sample	x (H/Pd)	a_H, b_H (Å)	c_H (Å)	Mean Diameter (nm)
(*) α-PdH_x	0.02	2.739	6.805	8.4 ± 0.13
(*) β-PdH_x/EG	0.09	2.764	6.765	5.6 ± 0.12
(*) β-PdH_x/SHEG	0.15	2.785	6.730	4.2 ± 0.11
(*) β-PdH_x/SEG	0.23	2.813	6.691	3.5 ± 0.09
(**) PdH_x	…	2.762	6.846	…

(*) Core unit cells are strained along the cubic $\{111\}_c$ plane deformed in the $<\bar{1}10>_c$ direction. Pseudo-hexagonal is used to describe the strained system with an α, β = 89.25° and γ = 120°.

(**) Shell unit cell is pseudo-hexagonal with a α, β = 81.65° and γ = 120°.
